# A Systematic Review and Meta-Analysis of the Diagnostic Value of Galectin-3 in Acute Coronary Syndrome

**DOI:** 10.3390/jcm13154504

**Published:** 2024-08-01

**Authors:** Michal Pruc, Zuzanna Gaca, Damian Swieczkowski, Jacek Kubica, Sagar Galwankar, Anna Salak, Lukasz Szarpak

**Affiliations:** 1 Department of Clinical Research and Development, LUX MED Group, 02-678 Warsaw, Poland; m.pruc@ptmk.org (M.P.); zuzanna.k.gaca@gmail.com (Z.G.);; 2 Department of Public Health, International European University, 03187 Kyiv, Ukraine; 3 Department of Toxicology, Faculty of Pharmacy, Medical University of Gdansk, 80-416 Gdansk, Poland; 4 Department of Cardiology and Internal Medicine, Nicolaus Copernicus University in Torun, Collegium Medicum in Bydgoszcz, 85-094 Bydgoszcz, Poland; jwkubica@gmail.com; 5 Department of Emergency, Florida State University College of Medicine, Emergency Medicine Residency Program, Sarasota Memorial Hospital, Sarasota, FL 32306, USA; gcsagar@yahoo.com; 6 Institute of Outcomes Research, Maria Sklodowska-Curie Medical Academy, 00-136 Warsaw, Poland; 7 Henry JN Taub Department of Emergency Medicine, Baylor College of Medicine, Houston, TX 77030, USA

**Keywords:** galectin-3, inflammation, biomarker, diagnosis, acute coronary syndrome

## Abstract

**Background/Objectives:** We investigated the potential diagnostic role of galectin-3 (Gal-3) in patients presenting with suspected acute coronary syndromes (ACS). **Methods:** We searched PubMed Central, Scopus, EMBASE, and the Cochrane Library from inception until 20 June 2024. We measured effect sizes using odds ratios (OR) with 95% CIs for dichotomous data and mean differences (MD) with CIs for continuous data. Random synthesis analysis was performed if I2 was less than 50% or Q test *p* values were less than 0.05. Otherwise, a fixed pooled meta-analysis was performed. **Results:** The meta-analysis includes 15 eligible studies. Gal-3 levels were substantially higher in the ACS group (12.84 ± 8.48 ng/mL) compared to the control group (7.23 ± 6.05 ng/mL; MD = 3.89; 95% CI: 2.83 to 4.95; *p* < 0.001). Gal-3 levels in acute myocardial infarction (AMI) and control groups differed (10.09 ± 8.16 vs. 4.64 ± 3.07 ng/mL, MD = 4.30; 95% CI: 0.41 to 8.18; *p* < 0.001). Statistical analysis revealed significant differences in Gal-3 levels between ST-elevated myocardial infarction (STEMI) and control groups (10.62 ± 7.34 vs. 5.54 ± 2.96 ng/mL; MD = 5.54; 95% CI: 3.12 to 7.97; *p* < 0.001). No significant differences were found between the non-ST-elevated myocardial infarction (NSTEMI) vs. control groups or patients with STEMI vs. patients with NSTEMI. **Conclusions:** Gal-3 may be beneficial for detecting acute coronary syndromes but not NSTEMI or differentiating between ACS types. This meta-analysis is promising, but further research is needed to prove Gal-3’s potential diagnostic value, exact cut-offs, and advantages over cardiospecific troponins. Gal-3 may be a useful diagnostic biomarker; however, more clinical trials are needed to prove its utility.

## 1. Introduction

Despite advances in diagnostics and therapeutic procedures in invasive cardiology, acute coronary syndrome (ACS) remains a significant clinical problem associated with a risk of premature death. A remarkable 50% reduction in age-standardised mortality rates has been observed over the past 20 years in developed countries. However, this positive trend is not mirrored in developing countries, where limited access to modern therapeutic methods impedes improvements in patient results [1].

To improve patient outcomes, there is a need for the development of new diagnostic models to identify patients at risk of ACS within the general population. Furthermore, it is crucial to identify patients at risk of adverse outcomes, such as rehospitalization or cardiovascular death after ACS [2]. Current risk assessment tools, such as the GRACE score, are based on multifactorial risk estimation and require detailed history taking, laboratory tests, or other medical procedures [3,4], which are costly and time-consuming. As a result, there is growing interest in biomarkers, especially metabolomics, that can facilitate accelerated diagnosis, identification of high-risk patients, and prognosis [5]. 

There are many diagnostic and prognostic biomarkers with potential use in cardiovascular diseases. A significant group of these are based on inflammatory markers, e.g., the neutrophil-to-lymphocyte ratio (NLR) and the platelet-to-lymphocyte ratio (PLR) [6,7,8]. Inflammatory markers are sometimes combined with other markers with a well-known role in the pathogenesis of atherosclerosis, as seen with the monocyte-to-HDL ratio [9]. Another extensively studied group of biomarkers are microRNAs (miRNAs). Zhang et al. demonstrated that increased expression of miR-361-5p is observed among patients with ACS, suggesting it as a new potential diagnostic parameter [10]. 

Galectin-3 (Gal-3) has recently emerged as a biomarker of significant interest, prompting renewed research and investigation of its potential applications. Gal-3 belongs to the β-galactosidase-binding lectins and is located mainly in the cytoplasm, although it can also be found in other cellular organelles, such as the nucleus. Produced mainly by cells of the immune system, Gal-3 is a pro-inflammatory factor involved in the process of apoptosis and activation of cytokine pathways [11]. In the context of cardiac remodeling, Gal-3 plays a significant role in the process of fibrosis, especially following acute inflammation [12]. Regardless of the effect of Gal-3 on cardiac fibrosis, Gal-3 contributes to pro-inflammatory damage to the vascular endothelium, triggering a cascade of inflammatory reactions. It also contributes to thrombus formation and the destabilization of the atherosclerotic plaque. All these processes are fundamental to the pathophysiology of atherosclerosis, ultimately leading to ACS [13]. Given the complex influence of Gal-3 on the mechanisms leading to the development of ACS, it is not surprising that interest in Gal-3 as a potential diagnostic and prognostic biomarker after ACS is growing [14,15]. 

The aim of this systematic review and meta-analysis is to explore the potential significance of galectin-3 levels as a diagnostic biomarker in patients with coronary syndrome.

## 2. Materials and Methods

The present meta-analysis was planned, conducted, and reported in accordance with the Preferred Reporting Items for Systematic Review and Meta-Analysis (PRISMA) guidelines [16]. The review protocol was registered in the PROSPERO International prospective register of systematic reviews, under registration number CRD42024553402, on 16 June 2024.

### 2.1. Search Strategy

A comprehensive literature search was conducted in databases including the PubMed/MEDLINE, Scopus, and EMBASE electronic databases (via the Ovid interface) and unpublished sources, such as the Cochrane Trial Registry and Clinicaltrials.gov, covering all records until 20 June 2024. The following search keywords were used: “galectin-3” OR “galectin 3” OR “Gal-3” OR “Gal 3” AND “acute coronary syndrome” OR “ACS” or “ST Segment Elevation Myocardial Infarction” OR “ST Elevated Myocardial Infarction” OR “ST-elevation MI” OR “STEMI” OR “non-ST elevation myocardial infraction” OR “NSTEMI” OR “myocardial Infarction” OR “unstable angina”. Furthermore, an additional investigation was conducted using Google Scholar. To identify additional relevant studies, reference lists of systematic reviews and relevant individual studies were examined. For studies with overlapping patient data, only the most recent publication was considered. 

### 2.2. Inclusion and Exclusion Criteria

The following inclusion criteria were used: (1) Patients: adult patients with ACS. (2) Articles discussing the levels of galectin-3 in various types of ACS. (3) Articles providing sufficient data to estimate standard mean differences (SMDs) among ACS types or the relative risk (RR) of mortality or major adverse cardiovascular events. (4) The study type included both RCTs and non-RCTs. (5) Full-text articles published in English.

The exclusion criteria included: (1) incomplete data or unpublished literature; (2) duplicate publications; (3) animal studies; and (4) review articles, letters, editorials, case reports/series, or conference abstracts.

### 2.3. Selection Process

We imported all articles identified through the search into EndNote X6 (Clarivate, London, UK), a reference management software, and removed duplicates. Two reviewers, M.P. and Z.G., independently screened titles and abstracts of the identified studies against the eligibility criteria. Then the potentially eligible studies underwent a full-text review—the same reviewers evaluated full texts for final eligibility, documenting reasons for exclusion. A third reviewer (L.S.) resolved any disagreements.

### 2.4. Data Collection

Two investigators independently performed data extraction. Extracted data included the following: study characteristics (first author, country, study design, study groups, and sample sizes), patient demographics (baseline characteristics), types of major adverse cardiovascular events (MACEs), mortality outcomes across different follow-up periods, and galectin-3 values. We used a pre-prepared data abstraction form in Microsoft Excel. We contacted the corresponding authors for further clarification or any unpublished relevant data.

### 2.5. Risk of Bias Assessment

Two researchers (M.P. and Z.G.) independently assessed the risk of bias using the Newcastle Ottawa Quality Assessment Scale (NOS) [17]. Each study received a score from 0 to 9 based on three categories: group selection (four items), comparability between groups (one item), and outcome (three items). Group selection, outcome, and exposure assessment categories could each receive a maximum of one star, while comparability could be awarded up to two stars. Thus, the maximum score was nine points, and a total score of six or above indicated high quality [17]. 

### 2.6. Data Analysis

Statistical analyses were performed using Stata 18.0 (Stata Corp., College Station, TX, USA) and RevMan 5.4 (Copenhagen: The Nordic Cochrane Centre, The Cochrane Collaboration, Copenhagen, Denmark). A two-sided *p* < 0.05 was considered statistically significant for all statistical analyses. For continuous outcomes, such as galectin-3 levels, effect sizes were expressed as mean differences (MD) with 95% CIs. Dichotomous variables were evaluated using odds ratios (OR). When continuous outcomes were reported as medians, ranges, and interquartile ranges, the formula described by Hozo et al. [18] was used to estimate means and standard deviations. The heterogeneity across all eligible studies was assessed using Cochran’s Q and I2 statistics. The I2 values of 25%, 50%, and 75% as cut-off points indicated low, moderate, and high degrees of heterogeneity, as per Cochrane’s guidelines [19]. We performed DerSimonian and Laird random effect models with inverse variance weights without any additional corrections [20]. For analyses including more than 10 studies, potential publication bias was evaluated using funnel plots and Begg’s or Egger’s tests. Sensitivity analysis was performed using the single study removal method to assess the robustness of the combined data.

## 3. Results

### 3.1. Results of Study Selection

**Figure 1:**
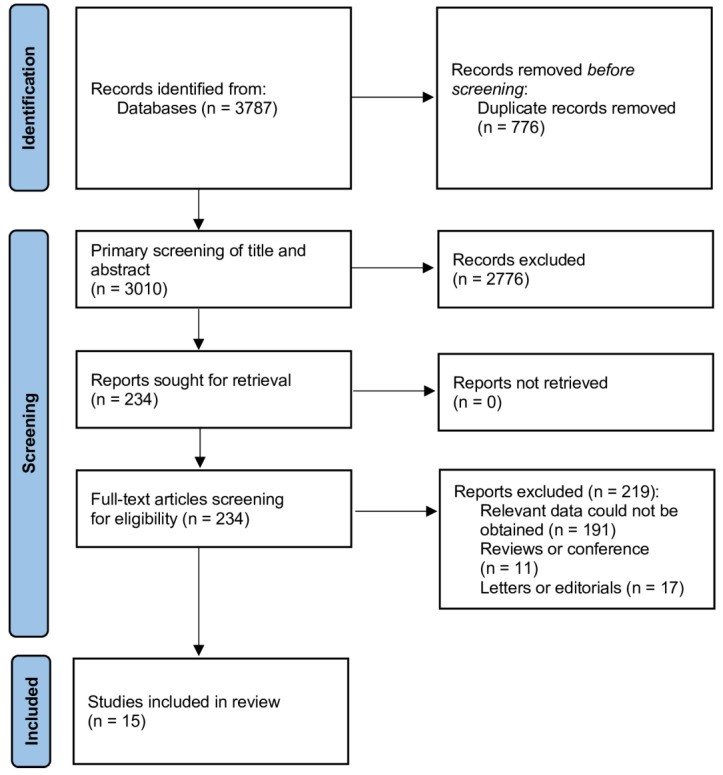
PRISMA flow diagram.

The flow diagram in Figure 1 shows the study’s selection method. We removed 777 duplicates from the 3786 results that the search yielded. After the title and abstract sieve, we excluded a total of 2776 studies, selecting 234 for full text review. This meta-analysis included a final total of 15 studies [21,22,23,24,25,26,27,28,29,30,31,32,33,34,35].

### 3.2. Descritpion of the Included Trials

**Figure 2:**
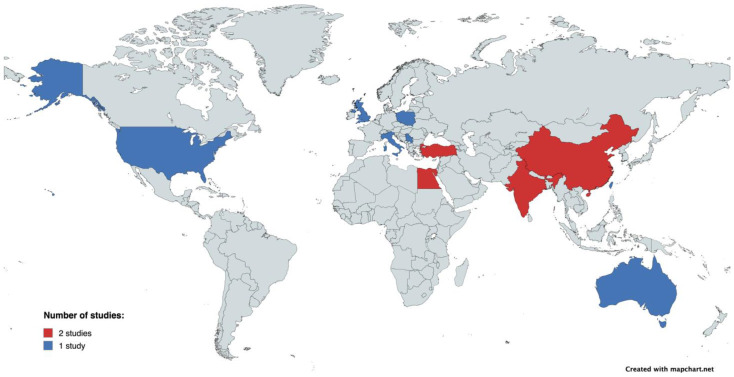
Worldwide distribution of studies included in the meta-analysis.

Table 1 summarizes the baseline characteristics of the included studies. A total of 2286 patients were involved in this analysis. We included 15 trials, published between 2012 and 2024. Among the 15 trials included in this meta-analysis, two studies were conducted in Egypt, Turkey, and India. The majority of the studies were prospective in design (*n* = 15). Most trials had two arms, while three arms were identified in four studies. The number of participants per arm varied between 17 and 196. Men constituted a significant proportion of participants included in the studies, ranging from 52.2% to 89.4%. The mean age of participants spanned from 34.6 to 67.4 years, with the notably younger cohort observed in the study by Winter et al. (2016) [35]. Table 1 illustrates considerable comorbidity among participants, including hypertension, diabetes mellitus, and hypercholesterolemia. A frequent reduction in left ventricular ejection fraction was also observed across the trials. Figure 2 displays a graphical representation of the origin of the studies included in the meta-analysis. Table 1 displays the NOS’s assessment of quality studies, with all studies receiving a high-quality rating.

### 3.3. Meta-Analysis Outcomes

**Figure 3:**
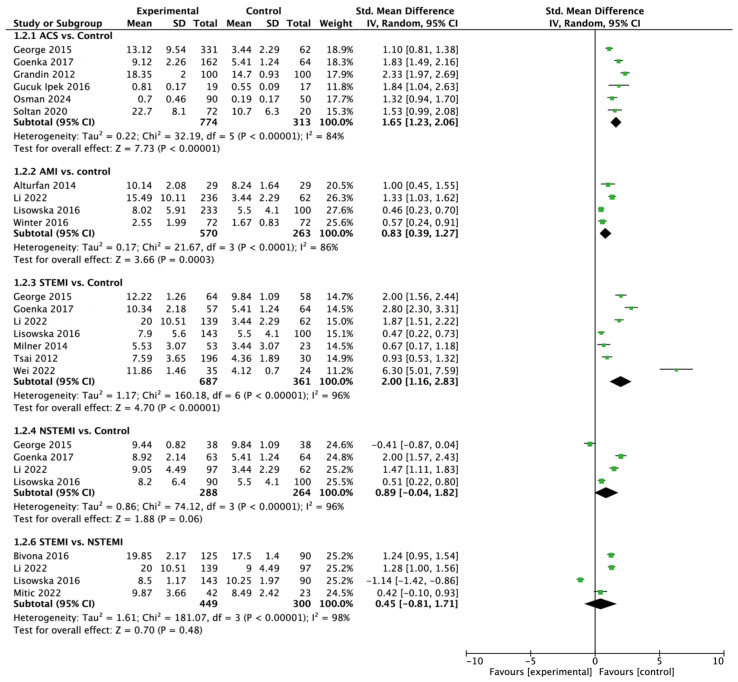
Forest plots of galectin-3 levels among different patient groups [21,22,23,24,25,26,27,28,29,30,31,32,33,34,35].

A pooled analysis of six studies showed that Gal-3 levels were statistically significantly higher in the ACS group (12.84 ± 8.48 ng/mL) compared to the control group (7.23 ± 6.05 ng/mL; MD = 3.89; 95% CI: 2.83 to 4.95; *p* < 0.001; Figure 3).

Four studies reported Gal-3 levels among acute myocardial infarction (AMI) and control groups. Pooled analysis of Gal-3 among AMI and control groups varied and amounted to 10.09 ± 8.16 vs. 4.64 ± 3.07 ng/mL, respectively (MD = 4.30; 95% CI: 0.41 to 8.18; *p* < 0.001).

Statistical analysis also showed statistically significant differences in Gal-3 levels between ST-elevated myocardial infarction (STEMI) and the control group (10.62 ± 7.34 vs. 5.54 ± 2.96 ng/mL; MD = 5.54; 95% CI: 3.12 to 7.97; *p* < 0.001).

In contrast, there were no statistically significant differences between the non-ST-elevated myocardial infarction (NSTEMI) and control group (8.81 ± 4.55 vs. 5.62 ± 3.43 ng/mL; MD = 2.84; 95% CI: −0.03 to 5.71; *p* = 0.05), or between patients with STEMI and patients with NSTEMI (13.35 ± 8.19 vs. 11.89 ± 4.75 ng/mL; MD—3.12; 95% CI: −0.34 to 6.58; *p* = 0.08).

The sensitivity analysis carried out did not show an impact on the results obtained.

## 4. Discussion

Our meta-analysis showed significantly higher levels of Gal-3 in the group of patients with ACS compared to the control group. A statistically significant difference was also found when comparing patients with AMI or STEMI to the control group. Additionally, there were no statistically significant differences between the NSTEMI and control or between patients with STEMI and patients with NSTEMI. The elevated Gal-3 values in the experimental groups may indicate increased inflammation and fibrosis following ACS.

The potential diagnostic and prognostic properties of the Gal-3 biomarker have been extensively studied in the context of cardiovascular diseases. Agnello et al. described the possible use of Gal-3 as a prognostic biomarker in heart failure (HF) following ACS, including in the prediction of cardiovascular death or HF following ACS [36]. Similar conclusions are also supported by other meta-analyses [37,38]. In the context of atrial fibrillation (AF), Gong et al. showed that elevated Gal-3 values were observed among patients with persistent AF [39]. While the current meta-analysis focuses mainly on the potential diagnostic utility of the Gal-3 biomarker, Chen et al. suggested that Gal-3 may also have potential prognostic value after acute heart failure, e.g., for predicting mortality and cardiovascular mortality [40]. Gal-3 has also shown predictive value in patients with aortic stenosis after transcatheter aortic valve replacement (TAVR) in terms of all-cause mortality [41]. Furthermore, elevated Gal-3 levels also correlate with an increased risk of cardiovascular events among patients with type 2 diabetes. Tan et al. defined a cardiovascular event as a composite endpoint including: first non-fatal myocardial infarction, non-fatal stroke, coronary revascularization, or cardiovascular-related death. In an observational study of 1495 patients, the authors demonstrated that Gal-3 levels were significantly elevated in patients who experienced a cardiovascular event during follow-up [42].

Gal-3 may offer certain advantages over cardiospecific troponins, which are widely used to diagnose and differentiate forms of ischemic heart disease and are a fundamental part of the diagnostic criteria for MI. Gal-3 may enable very early stratification of patients with an unfavourable prognosis or requiring intensified medical care or long-term secondary prevention [43]. However, it should be noted that Gal-3 is not a highly specific biomarker, which may significantly limit its application in the diagnosis utility of cardiovascular diseases. Syn et al. reported that elevated Gal-3 levels are also observed in chronic renal failure and may serve as a prognostic factor for disease progression [44], as well as a prognostic factor for renal failure following intensive care unit admission [45]. Some studies also indicate the potential diagnostic use of Gal-3 in certain cancers, such as pancreatic cancer [46]. King et al., showed that increased Gal-3 levels correlated with more severe depressive symptoms. However, this relationship is influenced by multiple variables such as multimorbidity, which may also be associated with increased depressive symptoms while also influencing increased Gal-3 values [47]. In chronic obstructive pulmonary disease (COPD), Gal-3 may predict symptom exacerbation [48]. These examples highlight the fact that the lack of specificity of Gal-3 may pose a significant challenge to its use as a potential diagnostic biomarker in cardiovascular diseases. The clinical superiority of Gal-3 compared to established biomarkers, such as B-type natriuretic peptide (BNP), has not been conclusively demonstrated. Both cardioselective troponins and Gal-3 are useful in cardiovascular risk stratification, but their prognostic value is not statistically significant after accounting for clinical factors [49].

In the context of pathophysiological mechanisms, it is worth noting that Gal-3 is known to induce inflammation, e.g., by activating macrophages; and inflammation itself plays an important role in promoting the development of atherosclerotic changes and atherosclerotic plaque instability. Thus, Gal-3 itself is sometimes referred to as a “biomarker of plaque progression and destabilization” [50,51]. Sygitowicz et al. noted that one reason for Gal-3’s impact on cardiovascular disease progression is its promotion of lipid accumulation in macrophages. Additionally, Gal-3’s pro-inflammatory properties contribute to endothelial dysfunction [52]. 

Gal-3’s impact on apoptosis also plays an important role in the pathophysiology of ACS. Gal-3 seems to be responsible for promoting apoptosis, which may initially increase the damage resulting from myocardial ischemia. Moreover, in the long term, the influence of Gal-3 on apoptosis may lead to increased fibrosis and, consequently, remodelling, contributing to the development of heart failure. Despite many studies, the pro- or anti-apoptotic properties of Gal-3 require further investigation, especially since inhibition or promotion of Gal-3 may potentially constitute a new target for drug development in cardiovascular diseases [53].

Regardless of its effect on fibrosis and apoptosis, Gal-3 may destabilise atherosclerotic plaque [36]. Gal-3 also contributes to increased oxidative stress [54]. It is worth noting that only a small number of compounds have been tested in the context of Gal-3 inhibition, mainly in animal models, including N-acetyllactosamine or LacDiNAc. TDI 139 is in early clinical trials for idiopathic pulmonary fibrosis. Zaborska et al. indicated that one of the main obstacles to advancing Gal-3 inhibitors from preclinical to clinical phases is the lack of appropriate animal models reflecting the complexity of HF [55]. 

The results of our meta-analysis are not entirely consistent with the pathophysiology discussed earlier. Gal-3 levels were higher in both patients with ACS and patients with MI, as compared to the control groups. While Gal-3 levels are elevated in patients with STEMI compared to controls, there were no statistically significant differences between the NSTEMI group and controls, as well as between patients with STEMI and patients with NSTEMI. These findings suggest that Gal-3 may be useful for diagnosing acute coronary syndromes, but it appears less effective for diagnosing NSTEMI or differentiating between all ACS types. 

When interpreting the results of this meta-analysis, several limitations of the study must be considered. First, subgroup analyses revealed significant heterogeneity. A high level of heterogeneity indicates that the trials included in the meta-analysis differ widely in terms of the magnitude of change of Gal-3 in patients with acute coronary syndromes. Hence, the high heterogeneity indicates that meta-analysis results should be interpreted with caution. Future research should aim to standardise methodologies and include more consistent trials to mitigate the above-mentioned limitations.

Furthermore, the definition of controls varied between the included studies. Another limitation is the lack of a clearly defined cut-off, a typical limitation of diagnostic biomarker meta-analyses. Further studies are necessary to validate the use of Gal-3 as a potential diagnostic biomarker, particularly in establishing cut-offs in prospective clinical trials as well as to determine its advantage over commonly used biomarkers of cardiovascular diseases, such as cardiospecific troponins.

Finally, we cannot perform a meta-analysis with specific diagnostic parameters such as ROC curves and C-statistics or estimate sensitivity and specificity since there is currently insufficient evidence to conduct such comprehensive analyses. Moreover, these indicators are particularly useful in assessing the clinical utility of diagnostic tests during their implementation in clinical practice, such as evaluating the clinical effectiveness of in vitro diagnostic medical devices. This meta-analysis is focused on exploring the potential diagnostic properties of Gal-3 and remains at the exploratory stage.

## 5. Conclusions

Our meta-analysis demonstrated significantly higher levels of Gal-3 in patients with ACS, AMI, or STEMI compared to controls, but with no differences between both NSTEMI compared to controls and STEMI vs. NSTEMI groups. These findings suggest that Gal-3 may have potential utility in diagnosing ACSs, but it appears less effective for diagnosing NSTEMI or differentiating between all ACS types. However, high heterogeneity in all subgroups indicates a lot of variability between studies, alongside the lack of standardised cut-off values, and should be considered when interpreting the results. Gal-3’s roles in inflammation, endothelial dysfunction, lipid accumulation in macrophages, apoptosis, and oxidative stress underscore its complex involvement in cardiovascular pathophysiology. Despite its diagnostic potential in cardiovascular diseases, the lack of specificity of Gal-3 limits its clinical use. While the findings of this meta-analysis are promising, additional studies are needed to explore Gal-3’s diagnostic utility, establish precise cut-offs, and confirm its advantages over established biomarkers such as cardiospecific troponins. Although the results suggest the potential value of Gal-3 as a diagnostic biomarker, further clinical studies are needed to confirm its utility and implementation in clinical practice.

## Figures and Tables

**Table 1:** Baseline characteristics of included trials.

Study	Country	Study Design	Study Group	No. of Patient’s	Age, Years	Sex, Male	Comorbidities				LVEF, %	NOS Score
							HTN, %	DM, %	HCL, %	Smoking, %		
Alturfan et al., 2014 [21]	Turkey	Prospective study	MI	29	NS	19 (65.5%)	NS	12 (41%)	18 (62%)	NS	NS	7
			Control	29	NS	21 (72.4%)	NS	0 (0%)	3 (10%)	NS	NS	
Bivona et al., 2016 [22]	Italy	Prospective study	STEMI	125	63.9 (14)	97 (77.6%)	NS	NS	NS	NS	47.5 (37.2–55)	8
			NSTEMI	90	67.4 (13.5)	64 (71.1%)	NS	NS	NS	NS	48 (40.5–55)	
George et al., 2015 [23]	India	Prospective study	STEMI	64	51.48 (10.6)	54 (84.4%)	26 (40.6%)	28 (43.8%)	30 (46.9%)	29 (45.3%)	44.18 (9.2)	8
			NSTEMI and UA	38	54.82 (10.03)	24 (63.2%)	15 (39.5%)	19 (50.0%)	15 (39.5%)	7 (18.4%)	53.3 (11.38)	
			Control	58	53.22 (9.45)	39 (67.2%)	28 (48.3%)	28 (48.3%)	23 (39.6%)	7 (12.5%)	52.82 (12.87)	
Goenka et al., 2017 [24]	India	Prospective study	STEMI	57	55.39 (11.88)	49 (86.0%)	15 (26.3%)	20 (35.1%)	5 (8.8%)	20 (35.1%)	46.00 (7.42)	9
			NSTEMI and UA	63	54.10 (9.76)	42 (66.7%)	32 (50.8%)	28 (44.4%)	9 (14.3%)	13 (20.6%)	57.48 (8.56)	
			Control	64	39.59 (12.94)	36 (56.2%)	13 (20.3%)	10 (15.6%)	4 (6.2%)	7 (10.9%)	60.13 (6.10)	
Grandin et al., 2012 [25]	USA	Case-control study	ACS	100	65.7 (10.9)	73 (73.0%)	68 (68.0%)	32 (32.0%)	NS	NS	NS	7
			Control	100	65.7 (10.9)	73 (73.0%)	54 (54.0%)	23 (23.0%)	NS	NS	NS	
Gucuk Ipek et al., 2016 [26]	Turkey	Prospective study	ACS	19	64.5 (7.6)	11 (57.9%)	7 (36.8%)	4 (21.1%)	4 (21.1%)	6 (31.6%)	NS	8
			Control	17	60.9 (7.6)	9 (52.9%)	4 (23.5%)	1 (5.9%)	6 (35.3%)	6 (35.3%)	NS	
Li et al., 2022 [27]	China	Retrospective study	STEMI	139	58.49 (13.19)	120 (89.4%)	76 (54.6%)	22 (15.8%)	3 (2.2%)	70 (50.4%)	58 (22–74)	9
			NSTEMI	97	61.95 (10.17)	82 (84.5%)	60 (75.9%)	22 (22.7%)	3 (3.1%)	37 (38.1%)	62 (30–80)	
			Control	62	60.35 (10.36)	39 (62.9%)	26 (41.9%)	8 (12.9%)	1 (1.6%)	23 (37.1%)	65 (48–75)	
Lisowska et al., 2016 [28]	Poland	Prospective cohort study	STEMI	143	63.3 (9.9)	116 (81.1%)	97 (67.8%)	41 (28.7%)	52 (36.4%)	96 (67.1%)	44.5 (9.8)	9
			NSTEMI	90	63.5 (11.8)	62 (68.9%)	51 (56.7%)	12 (13.3%)	37 (41.1%)	57 (63.3%)	48.6 (10.5)	
			Control	100	61.5 (7.9)	67 (66.3%)	0 (0.0%)	0 (0.0%)	0 (0.0%)	28 (28.0%)	55.0 (12.5)	
Milner et al., 2014 [29]	UK	Prospective study	STEMI	53	60.6	39 (73.8%)	24 (45.3%)	5 (9.4%)	19 (35.8%)	30 (56.6%)	NS	7
			Control	23	54.1	12 (52.2%)	8 (34.8%)	0 (0.0%)	7 (30.4%)	9 (39.1%)	NS	
Mitic et al., 2022 [30]	Serbia	Prospective study	STEMI	42	63.81 (9.84)	27 (64.3%)	NS	NS	NS	15 (35.7%)	50 (46.5–55.0)	8
			NSTEMI	23	64.00 (9.60)	18 (78.3%)	NS	NS	NS	6 (26.1%)	54 (50.0–58.0)	
Osman et al., 2024 [31]	Egypt	Prospective study	ACS	90	59.4 (6.49)	59 (65.6%)	48 (53.3%)	42 (44.6%)	44 (48.9%)	50 (55.6%)	50.86 (5.13)	8
			Control	50	57.9 (7.83)	33 (66.0%)	23 (46.0%)	11 (22.0%)	24 (48.0%)	17 (34.0%)	59.46 (6.3)	
Soltan et al., 2020 [32]	Egypt	Prospective study	ACS	72	56.3 (12.7)	54 (75.0%)	30 (41.7%)	33 (45.8%)	9 (12.5%)	56 (77.8%)	NS	7
			Control	20	51.6 (7.7)	14 (70.0%)	10 (50.0%)	7 (35.0%)	3 (15.0%)	12 (60.0%)	NS	
Tsai et al., 2012 [33]	Taiwan	Prospective study	AMI	196	62.2 (12.1)	126 (82.7%)	109 (55.6%)	67 (34.2%)	NS	115 (58.7%)	NS	8
			Control	30	62.1 (7.1)	23 (76.7%)	0 (0.0%)	0 (0.0%)	NS	0 (0.0%)	NS	
Wei et al., 2022 [34]	China	Prospective study	STEMI	35	57.25 (10.58)	22 (62.9%)	11 (31.4%)	NS	NS	16 (45.7%)	49.37 (7.97)	8
			Control	24	57.84 (10.32)	15 (62.5%)	6 (25.0%)	NS	NS	8 (33.3%)	58.33 (1.58)	
Winter et al., 2016 [35]	Austria	Multicenter case-control study	MI	72	35.6 (4.3)	64 (89%)	30 (42%)	19 (26%)	NS	55 (76%)	NS	8
			Control	72	34.6 (4.6)	64 (89%)	6 (8%)	4 (6%)	NS	37 (51%)	NS	

Legend: MI = myocardial infarction; STEMI = ST-elevation myocardial infarction; NSTEMI = non-ST-elevation myocardial infarction; UA—unstable angina; ACS—acute coronary syndrome; DM = diabetes melitus; HCL = hypercholesterolemia; HTN = hypertension; NS = not specified; LVEF = left ventricle ejection fraction; NOS = Newcastle–Ottawa Scale.

## Data Availability

The data that support the findings of this study are available on request from the corresponding author (L.S.).
